# Inertial Measurement Unit-Based Romberg Test for Assessing Adults With Vestibular Hypofunction

**DOI:** 10.1109/JTEHM.2023.3334238

**Published:** 2023-11-17

**Authors:** Kuan-Chung Ting, Yu-Chieh Lin, Chia-Tai Chan, Tzong-Yang Tu, Chun-Che Shih, Kai-Chun Liu, Yu Tsao

**Affiliations:** Department of Otolaryngology-Head and Neck SurgeryTaipei Veterans General Hospital 46615 Taipei 11217 Taiwan; Institute of Clinical Medicine, National Yang Ming Chiao Tung University Taipei 11221 Taiwan; Department of Biomedical EngineeringNational Yang Ming Chiao Tung University Taipei 11221 Taiwan; Research Center for Information Technology InnovationAcademia Sinica 38017 Taipei 11529 Taiwan; School of MedicineNational Yang Ming Chiao Tung University Taipei 11221 Taiwan; Division of Cardiovascular SurgeryTaipei Municipal Wanfang Hospital Taipei 11608 Taiwan; Taipei Heart Institute, Taipei Medical University 38032 Taipei 11013 Taiwan

**Keywords:** Vestibular hypofunction, Romberg test, tandem Romberg test, wearable sensor, inertial measurement units

## Abstract

This work aims to explore the utility of wearable inertial measurement units (IMUs) for quantifying movement in Romberg tests and investigate the extent of movement in adults with vestibular hypofunction (VH). A cross-sectional study was conducted at an academic tertiary medical center between March 2021 and April 2022. Adults diagnosed with unilateral vestibular hypofunction (UVH) or bilateral vestibular hypofunction (BVH) were enrolled in the VH group. Healthy controls (HCs) were recruited from community or outpatient clinics. The IMU-based instrumented Romberg and tandem Romberg tests on the floor were applied to both groups. The primary outcomes were kinematic body metrics (maximum acceleration [ACC], mean ACC, root mean square [RMS] of ACC, and mean sway velocity [MV]) along the medio-lateral (ML), cranio-caudal (CC), and antero-posterior (AP) axes. A total of 31 VH participants (mean age, 33.48 [SD 7.68] years; 19 [61%] female) and 31 HCs (mean age, 30.65 [SD 5.89] years; 18 [58%] female) were recruited. During the eyes-closed portion of the Romberg test, VH participants demonstrated significantly higher maximum ACC and increased RMS of ACC in head movement, as well as higher maximum ACC in pelvic movement along the ML axis. In the same test condition, individuals with BVH exhibited notably higher maximum ACC and RMS of ACC along the ML axis in head and pelvic movements compared with HCs. Additionally, BVH participants exhibited markedly increased maximum ACC along the ML axis in head movement during the eyes-open portion of the tandem Romberg test. Conversely, no significant differences were found between UVH participants and HCs in the assessed parameters. The instrumented Romberg and tandem Romberg tests characterized the kinematic differences in head, pelvis, and ankle movement between VH and healthy adults. The findings suggest that these kinematic body metrics can be useful for screening BVH and can provide goals for vestibular rehabilitation.

## I. Introduction

Vestibular hypofunction (VH) can cause functional declines in vision, gait, and balance [1], [2], [3]. Individuals with VH may have trouble maintaining stability during tasks involving standing, as postural balance requires the integration of visual, somatosensory, and vestibular signals and is regulated by the central nervous system [4].

Various physical tests have been developed to examine the role of vestibular function in standing posture control. A typical physical test is the Romberg test, which evaluates static balance while the subject is standing with the eyes open and closed. If individuals are unstable while standing (e.g., exhibit body sways and movement), the test is considered positive. Romberg’s test is part of neurological examinations in the outpatient department or general ward [5], [6]. The tandem Romberg test is a modified version of the Romberg test commonly used in diving medicine [7], [8], [9]. Individuals with vestibular problems cannot complete the tandem Romberg test; therefore, this assessment can be a diagnostic tool for evaluating vestibular disorders [10]. However, the sensitivity and reliability of these tests are limited because of issues related to observer bias [6] and age-related changes [11].

A review of the literature indicates that numerous studies have been performed on the role of vestibular cues in static balance control using advanced sensor technologies [12]. For example, force plates or moving platform posturography have been used to measure imbalance due to vestibular deficits using different motion features (e.g., static tilt angles, peak velocity, and duration) in different directions and with various body parts (e.g., head and trunk). However, moving platform posturography is expensive, bulky, and unavailable in primary care. Moreover, this technology assesses overall trunk control but is limited in tracking individual body parts at a fine-grained level. Detailed posture information is essential because it is associated with body coordination and offers richer kinematic metrics for clinical evaluation.

Numerous studies have contributed valuable insights into the assessment of balance, underscoring the potential of innovative devices for evaluating vestibular function. Janc et al. compared head movement tests using force plate and accelerometer-based posturography, highlighting the ability of both devices to differentiate between patients with balance problems and healthy individuals [13]. Rosiak et al. evaluated the utility of the MediPost Mobile Posturography Device in assessing patients with a unilateral vestibular disorder, reporting high sensitivity and specificity in distinguishing between healthy individuals and those with a vestibular deficit [14]. Zobeiri et al. focused on head movement kinematics during functional gait assessment in patients undergoing vestibular schwannoma resection; microelectromechanical systems (MEMS) were used to record and analyze head movements [15]. The study highlights the impact of vestibular damage and compensation on postural control during gait tasks and emphasizes the importance of quantifying kinematics using MEMS technology in assessing balance and compensation. These studies provide valuable insights into balance assessment techniques and emphasize the potential of novel devices for evaluating vestibular function.

In recent years, there has been growing interest in the use of wearable inertial measurement units (IMUs) for motion analysis [16], [17], [18], [19] and balance tests [20]. IMUs offer several advantages, including portability, wireless connectivity, and the ability to provide real-time information on body movements in various settings [17]. However, the majority of previous research utilizing IMUs focused on dynamic balance assessments, with limited exploration of static balance assessments. For instance, Paul et al. utilized multiple IMUs to measure head–trunk kinematic abnormalities during dynamic gait tests in patients with unilateral vestibular loss, observing reduced head-turn amplitude and velocities [21]. In another study, researchers successfully differentiated between patients with chronic vestibular loss and healthy controls (HCs) by analyzing IMU data on gait stability [3]. Furthermore, researchers have examined the utility of IMU-based approaches for objective posture stability assessment in various conditions, such as cerebellar ataxia [22] and frailty [23]. To the best of our knowledge, wearable IMUs have not been previously applied to quantify static balance in individuals with vestibular deficits—particularly those with unilateral vestibular hypofunction (UVH) or bilateral vestibular hypofunction (BVH). This highlights the novelty and significance of our study; we aimed to fill the aforementioned gap by investigating the feasibility and potential benefits of IMU-based assessments in static balance testing.

In the present study, we investigated the advantages of IMUs in instrumented Romberg and tandem Romberg tests in terms of quantification of kinematic characteristics and the ability to detect abnormal body motion in adults with VH. The main contributions of the study are as follows. (1) Quantification of balance measures: Traditional Romberg and tandem Romberg tests rely on visual observation to assess patients’ balance, which can be subjective and vary across different studies. It is worth noting that positive results from these tests can vary among different sources. In contrast, in our study, IMUs were utilized to quantify the sway of various body segments, providing numerical values that objectively indicated the degree of body sway. This quantitative approach allowed more accurate and consistent assessment of balance performance. By quantifying the kinematic characteristics of different body parts, we aimed to clarify VH and its impact on balance control. (2) Comprehensive assessment: While clinical tests (e.g., portable force plate) may be easy to set up and inexpensive, they primarily focus on visually observing the changes in the body’s center of gravity. In contrast, IMUs provide the ability to evaluate a broader range of body segments, including the head, pelvis, and ankles. This comprehensive assessment of multiple body parts allows more thorough evaluation of balance control in individuals with VH. In comparison, a portable force plate primarily assesses changes in overall body weight distribution, which provides valuable information but may not capture the detailed kinematic characteristics offered by IMUs. According to these considerations, the main hypothesis of our study was that the IMU-based Romberg test and tandem Romberg test are feasible for obtaining advanced balance control metrics of different body parts in patients with VH, even when observational testing is not. By leveraging the quantification capabilities of IMUs and assessing various body movements, we obtained valuable insights into the assessment of balance impairments associated with VH.

## II. Methods

### A. Participants

This cross-sectional study was completed in an academic tertiary medical center, and data were collected between March 2021 and April 2022. We recruited adults who were 20 to 49 years of age. Individuals in outpatient departments who were diagnosed with UVH or BVH were enrolled in the VH group. Healthy participants without a history of dizziness or vertigo were recruited from community or outpatient clinics as HCs. The inclusion criteria for all the participants were as follows: no history of central vertigo, trauma, cancer, or neurological diseases (such as parkinsonism or stroke); normal activities of daily living without visual, musculoskeletal, or neurological problems; and no sedative or anti-vertigo medication use for 2 days before the test. The Institutional Review Board of Taipei Veterans General Hospital approved the study. All participants provided written informed consent.

### B. Criteria for Vestibular Hypofunction

Videonystagmography (VNG) was used to diagnose VH. UVH was defined as ≥25% weakness in the caloric VNG test [3]. BVH was defined as the sum of the bithermal maximum peak slow phase velocity being $ < 6~^{\circ }$/s on each side [24].

### C. Study Design and Experimental Protocol

**FIGURE 1.**
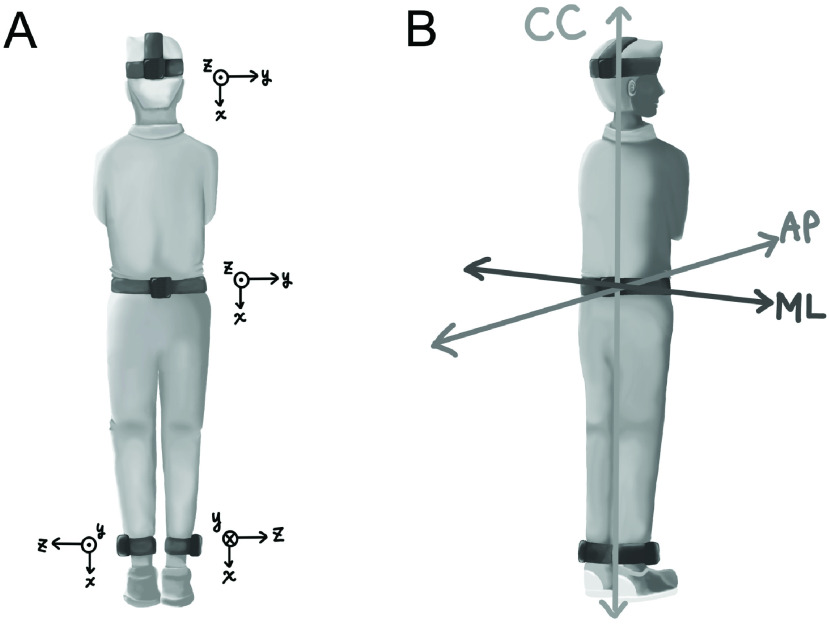
Illustration of the sensor placement and data preprocessing. For analysis, the three axes (x, y, z) of the four sensors (A) were converted into three anatomical axes: the antero-posterior (AP), medio-lateral (ML), and cranio-caudal (CC) axes (B).

Participants performed the Romberg and tandem Romberg tests on the floor while wearing IMUs (Opal sensors, version 2.0; Motion Studio software, version 2.0; APDM, Inc.) attached to specific body locations. The IMUs were secured on the head (occipital cranial bone), pelvis (L4-L5 level), and both ankles (above the lateral malleolus) using the straps provided by the manufacturer (Fig. 1A). These IMUs, which consisted of triaxial accelerometers, gyroscopes, and magnetometers, were used to collect three-dimensional (3D) linear accelerations and angular velocities. In particular, this study focused on analyzing body movements using 3D linear accelerations. Each IMU had dimensions of $48.5\times 36.5\times13.5$ mm^3^ and weighed 22 g. The sampling rate was 128 Hz, and the battery life of the sensor allowed 8 h of continuous data logging.

The Romberg test involved having the participant stand upright on a flat and firm surface with feet together and arms crossed; the participants were instructed to maintain this standing posture with their eyes open for 30 s and then with their eyes closed for another 30 s. In the tandem Romberg test, participants were asked to stand with one foot directly in front of the other foot (heel to toe). They chose which foot to place in front. Then, they were instructed to stand with their arms crossed and to maintain this posture with their eyes open for 30 s and then with their eyes closed for another 30 s. The test was terminated if the participants showed postural instability with a risk of falling.

### D. Data Preprocessing and Kinematic Parameter Extraction

**FIGURE 2.**
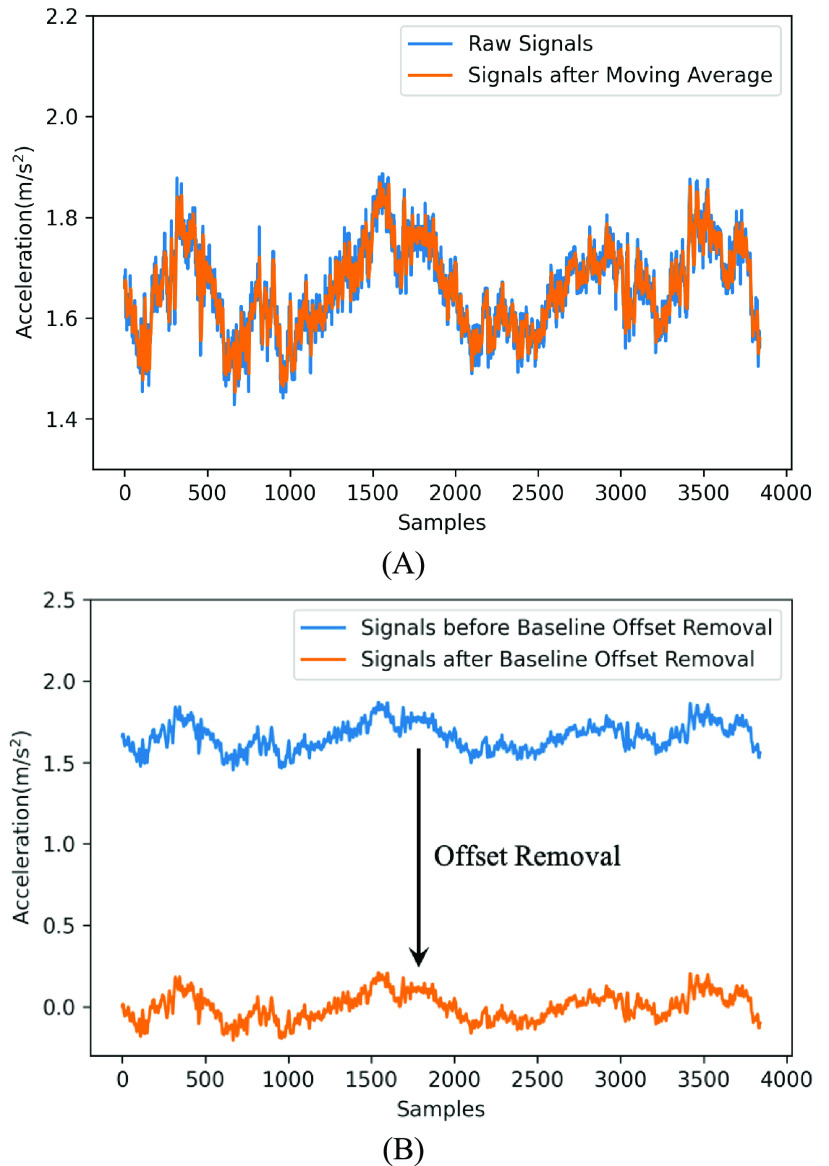
Example signals after the moving average filter (A) and the baseline offset removal (B).

The data preprocessing consisted of two stages. First, to reduce the amount of noise and artifacts caused by muscle vibration, various moving average approaches have been utilized for movement analysis, such as the moving average and Gaussian-weighted moving average [25]. We applied the standard moving average filter to smooth and denoise motion data in this study. A simple moving average was obtained by calculating the arithmetic mean of the determined period of signals, where the length of the period is determined as 5 data points. An example signal after the application of the moving average filter is shown in Figure 2A.

Then, an initial calibration was employed to the filtered data to remove the baseline offset. This is because the sensor placement and individual differences may cause sensor tilting and an initial baseline offset to the sensing signals. To remove such measurement error, the average of the complete signals over the test was subtracted from the entire data series and to generates the preprocessed data. This approach can efficiently remove the static baseline offset [26]. An example signal after the baseline offset removal is shown in Figure 2B.

After data preprocessing, kinematic parameters were extracted from the preprocessed segments of different tests. The IMU features were extracted along three axes from four sensor locations to analyze the performance, including significant differences between participants with VH and HCs. A verticalized frame aligned to the gravity vector was used to guarantee a repeatable reference system. The three axes of the four sensors were converted into three anatomical axes, i.e., the antero-posterior (AP), medio-lateral (ML), and cranio-caudal (CC) axes, for analysis (Fig. 1B). The ankle parameters were averaged between the two ankles.

The metrics of interest were the maximum acceleration (ACC), mean ACC, mean sway velocity (MV), and root mean square (RMS) of ACC. The RMS of ACC is a measure of the variation in acceleration relative to the mean [27], [28]. The following metrics were used.

• **Maximum ACC:** the maximum of accelerations in the AP, ML, and CC directions of the head, pelvis, or ankles (m/s^2^).
• **Mean ACC:** the average of accelerations in the AP, ML, and CC directions of the head, pelvis, or ankles (m/s^2^).
• **RMS of ACC:** the root mean square of accelerations in the AP, ML, and CC directions of the head, pelvis, or ankles (m/s^2^).
• **MV:** the integration of accelerations in the AP, ML, or CC directions of the head, pelvis, or ankles (m/s).

These kinematic metrics have been widely used in static balance analyses [29], [30], [31], [32], [33], [34]. A total of 144 parameters were analyzed, including 36 parameters [four types of metrics (maximum ACC, mean ACC, RMS of ACC, MV) $\times $ three anatomical axes (AP, ML, CC) $\times $ three body parts (head, pelvis, ankles)] shared by the Romberg and tandem Romberg tests in the eyes-open and eyes-closed portions.

### E. Statistical Analysis

**FIGURE 3.**
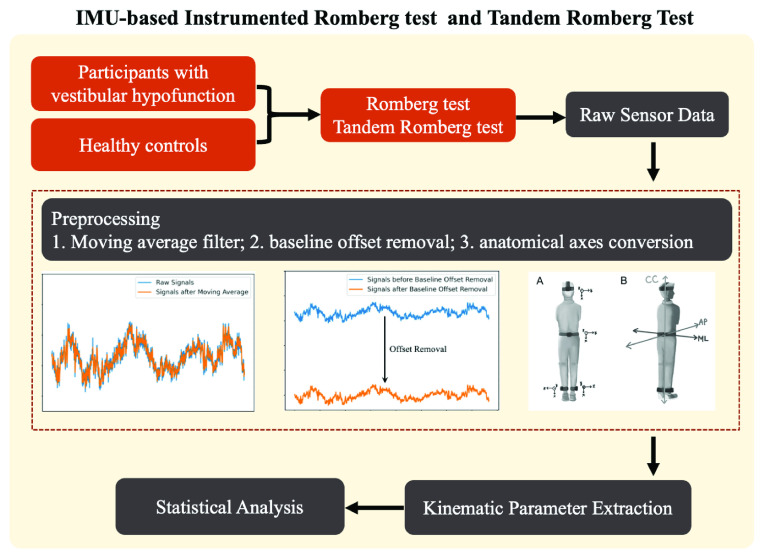
Framework of the IMU-based instrumented Romberg test and tandem Romberg test.

Each parameter is expressed in terms of the mean and standard deviation (SD). Group comparisons of categorical data, including age, sex, body mass index (BMI), and parameters, were analyzed using independent-sample $t$ tests. Subgroups were initially compared using one-way analysis of variance (ANOVA) tests. When significant differences were observed in the ANOVA results, we proceeded with the Bonferroni correction method to assess the relationships between multiple dependent variables simultaneously. This correction was aimed at maintaining an appropriate familywise error rate given the potential for multiple pairwise comparisons. We applied a correction factor of 12 because our primary objective was to determine which anatomical axes and kinematic metrics effectively differentiated between the groups under specific sensor placements and established balance tests. This choice was based on the consideration of three anatomical axes (AP, ML, CC) and four statistical features (maximum ACC, mean ACC, RMS of ACC, MV). Subsequently, for post hoc comparisons, we conducted Scheffe-corrected $t$ tests on these variables with corrected $p$-values that remained significant after Bonferroni correction. A $p$-value of < 0.05 was regarded as statistically significant. All statistical analyses were performed using Statistical Package for Social Sciences (SPSS) software (v.23 for Mac, SPSS Inc., Chicago, IL). The framework of the proposed IMU-based instrumented Romberg and tandem Romberg tests is shown in Figure 3.

## III. Results

### A. Participant Characteristics

Thirty-one participants with VH (mean age, 33.48 [SD 7.68] years; 19 [61%] female; 20 [65%] with BVH) and 31 HCs (mean age, 30.65 [SD 5.89] years; 18 [58%] female) were recruited. No group differences were found in terms of age, sex, or BMI. The participant demographic characteristics are summarized in Table 1.

**TABLE 1:** Demographic Characteristics (n = 62)

Characteristic	VH group ( $\text{n}=31$)	HC group ( $\text{n}=31$)	$p$-value	BVH group ( $\text{n}=20$)	UVH group ( $\text{n}=11$)	$p$-value^a^
Age, years	0.138					0.714
Mean±SD	33.48±7.68	30.65±5.89		33.10±7.83	34.18±7.70	
Range	22–46	24–46		22–46	25–45	
Sex (F:M)	1.000					1.000
Female	19 (61%)	18 (58%)		12 (60%)	7 (64%)	
Male	12 (39%)	13 (42%)		8 (40%)	4 (36%)	
BMI, kg/m^2^						
Mean±SD	23.26±2.01	22.13±3.34	0.112	22.97±1.87	23.80±2.22	0.275

^a^ Independent two sample t test; VH = vestibular hypofunction, HC = healthy control, BVH = bilateral vestibular hypofunction, UVH = unilateral vestibular hypofunction, SD = standard deviation, BMI = body mass index, BVH = bilateral vestibular hypofunction, UVH = unilateral vestibular hypofunction.

### B. VH and HC Groups Significantly Differed in Performance on Romberg Test

To determine whether participants with VH differed from HCs in terms of performance on the Romberg and tandem Romberg tests, we first aimed to determine group differences in the eyes-open portion of the Romberg test with independent-sample $t$ tests. As shown in Table 2, in the Romberg test, no differences between the two groups were found in the eyes-open portion. Compared with HCs, participants with VH exhibited a trend toward larger head, pelvis, and ankle movements with higher maximum ACC, mean ACC, RMS of ACC, and MV along the ML axis when standing with their eyes closed. After the Bonferroni correction was applied to the $p$-values, statistical significance remained for the head and pelvic movements along the ML axis of maximum ACC, as well as for head movement in the RMS of ACC (Table 2). This outcome indicates that the instrumented eyes-closed portion of the Romberg test not only successfully differentiated between participants with VH and HCs but also maintained its discriminatory power even when accounting for multiple comparisons.

**TABLE 2:** Significant Differences in Parameters of the Romberg Test Between hcs and Participants With VH

	VH group ( $\text{n}=31$) (mean±SD)	HC group ( $\text{n}=31$) (mean±SD)	$p$-value^a^	Bonferroni-corrected $p$-value	Effect Size (Cohen’s d)	95% CI
Romberg test (eyes open)						
–	–	–	–			–
Romberg test (eyes closed)						
Head						
Medio-lateral axis						
ACC (maximum), m/s2	0.48±0.27	0.30±0.09	0.001	0.012	0.89	0.074–0.279
ACC (mean), m/s2	0.10±0.05	0.07±0.02	0.006	0.072	0.74	0.009–0.050
ACC (RMS), m/s2	0.13±0.07	0.09±0.03	0.003	0.036	0.79	0.014–0.065
MV, m/s	0.98±0.51	0.71±0.25	0.011	0.132	0.68	0.066–0.479
Pelvis						
Medio-lateral axis						
ACC (maximum), m/s2	0.33±0.18	0.22±0.08	0.003	0.036	0.80	0.039–0.181
ACC (mean), m/s2	0.07±0.04	0.05±0.02	0.007	0.084	0.71	0.006–0.035
ACC (RMS), m/s2	0.09±0.04	0.06±0.02	0.005	0.06	0.75	0.008–0.044
MV, m/s	0.68±0.35	0.47±0.16	0.005	0.06	0.76	0.066–0.343
Ankles						
Medio-lateral axis						
ACC (maximum), m/s2	0.29±0.16	0.22±0.09	0.038	0.456	0.54	0.004–0.135
ACC (mean), m/s2	0.06±0.03	0.05±0.02	0.034	0.408	0.56	0.001–0.030
ACC (RMS), m/s2	0.08±0.04	0.06±0.02	0.034	0.408	0.55	0.001–0.037
MV, m/s	0.61±0.34	0.47±0.20	0.043	0.516	0.73	0.004–0.283

^a^ Independent two sample $t$ test, VH = vestibular hypofunction, HCs = healthy controls, ACC = acceleration, RMS = root mean square, MV = mean sway velocity, SD = standard deviation, – = not reported because of no significant difference

### C. Differences in Performance on Tandem Romberg Test Between VH and HC Groups

In the eyes-open portion of the tandem Romberg test, participants with VH exhibited a tendency toward higher maximum ACC, mean ACC, RMS of ACC, and MV along the ML axis compared with HCs. Additionally, participants with VH exhibited increased ankle movement with higher MV along the AP axis. However, after the Bonferroni correction was applied to the $p$-values, none of these parameters remained statistically significant.

Similarly, as shown in Table 3, in the eyes-closed portion of the tandem Romberg test, participants with VH exhibited head movement along the ML axis with higher mean ACC, RMS of ACC, and MV compared with HCs. Overall, there were fewer significant differences in movement metrics in the eyes-closed portion of the tandem Romberg test than in the eyes-open portion, with significant differences primarily observed in head movement. However, when we accounted for multiple comparisons using the Bonferroni correction, none of these differences remained statistically significant (Table 3). Despite these findings, it is important to note that the instrumented eyes-open portion of the tandem Romberg test initially exhibited discriminatory power between participants with VH and HCs.

**TABLE 3:** Significant Differences in Parameters of the Tandem Romberg Test Between HCs and Participants With VH

	VH group ( $\text{n}=31$) (mean±SD)	HC group ( $\text{n}=31$) (mean±SD)	$p$-value^a^	Bonferroni-corrected $p$-value	Effect Size (Cohen’s d)	95% CI
Tandem Romberg test (eyes open)						
Head						
Medio-lateral axis						
ACC (maximum), m/s2	0.75±0.50	0.47±0.19	0.007	0.084	0.723	0.080–0.470
ACC (mean), m/s2	0.15±0.07	0.11±0.04	0.010	0.12	0.685	0.010–0.070
ACC (RMS), m/s2	0.20±0.10	0.14±0.05	0.007	0.084	0.722	0.016–0.097
MV, m/s	1.54±0.77	1.13±0.38	0.011	0.132	0.676	0.099–0.718
Pelvis						
Medio-lateral axis						
ACC (maximum), m/s2	0.38±0.21	0.28±0.09	0.017	0.204	0.631	0.019–0.184
ACC (mean), m/s2	0.08±0.04	0.06±0.02	0.011	0.132	0.679	0.005–0.034
ACC (RMS), m/s2	0.10±0.05	0.08±0.02	0.010	0.12	0.687	0.006–0.044
MV, m/s	0.81±0.41	0.61±0.18	0.014	0.168	0.649	0.043–0.366
Ankles						
Antero-posterior axis						
MV, m/s	0.89±0.43	0.67±0.41	0.047	0.564	0.516	0.003–0.431
Tandem Romberg test (eyes closed)						
Head						
Medio-lateral axis						
ACC (mean), m/s2	0.32±0.22	0.21±0.08	0.018	0.216	0.674	0.020–0.202
ACC (RMS), m/s2	0.44±0.32	0.28±0.13	0.024	0.288	0.639	0.022–0.293
MV, m/s	3.24±2.35	2.08±0.76	0.020	0.24	0.666	0.201–2.130

^a^ Independent two sample $t$ test, VH = vestibular hypofunction, HCs = healthy controls, ACC = acceleration, RMS = root mean square, MV = mean sway velocity, SD = standard deviation, – = not reported because of no significant difference

### D. Subgroup Analysis: BVH VS. UVH VS. HCs

To comprehensively explore the distinctions within the VH group, we divided it into two subgroups: BVH and UVH participants, which were analyzed alongside HCs. We conducted comparisons between BVH and UVH, between UVH and HCs, and between BVH and HCs. The results of these subgroup analyses are presented in Tables 4 and 5.

**TABLE 4:** Subgroup Differences on the Romberg Test

	One-way ANOVA								Post hoc comparisons^b^		
	BVH group ( $\text{n}=20$) (mean±SD)		UVH group ( $\text{n}=11$) (mean±SD)	HC group ( $\text{n}=31$) (mean±SD)	$F$	$p$-value	$p$-value^a^	$\eta ^{2}$	BVH vs. UVH groups	UVH vs. HC groups	BVH vs. HC groups
									$p$-value	$p$-value	$p$-value
Romberg test (eyes closed)											
Head											
Medio-lateral axis											
ACC (maximum), m/s^2^	0.51±0.30		0.43±0.20	0.30±0.09	6.725	0.002	0.028	0.186	0.558	0.211	0.003
ACC (mean), m/s^2^	0.11±0.06		0.09±0.04	0.07±0.02	5.277	0.008	0.094	0.152			
ACC (RMS), m/s^2^	0.14±0.07		0.11±0.05	0.09±0.03	6.060	0.004	0.048	0.170	0.333	0.467	0.004
MV, m/s	1.06±0.57		0.83±0.36	0.71±0.25	4.766	0.012	0.063	0.139			
Pelvis											
Medio-lateral axis											
ACC (maximum), m/s^2^	0.37±0.20		0.27±0.11	0.22±0.08	7.063	0.002	0.021	0.193	0.156	0.627	0.002
ACC (mean), m/s^2^	0.08±0.04		0.06±0.03	0.05±0.02	5.560	0.006	0.074	0.159			
ACC (RMS), m/s^2^	0.10±0.05		0.07±0.03	0.06±0.02	6.016	0.004	0.050	0.169	0.221	0.639	0.004
MV, m/s	0.73±0.38		0.58±0.26	0.47±0.16	5.753	0.005	0.145	0.163			
Ankles											
Medio-lateral axis											
ACC (maximum), m/s^2^	0.32±0.18		0.24±0.12	0.22±0.09	3.579	0.034	0.409	0.108			
ACC (mean), m/s^2^	0.07±0.04		0.05±0.02	0.05±0.02	4.368	0.017	0.204	0.129			
ACC (RMS), m/s^2^	0.09±0.05		0.06±0.03	0.06±0.02	4.335	0.018	0.210	0.128			
MV, m/s	0.67±0.37		0.51±0.25	0.47±0.20	3.455	0.038	0.457	0.105			
Cranio-caudal axis											
ACC (maximum), m/s	0.15±0.11		0.08±0.04	0.09±0.04	4.724	0.013	0.150	0.138			

^a^ =Bonferroni-corrected $P$ value, $^{}\eta ^{2}$ = eta square,

^b^ = Scheffe-corrected post hoc $t$ test, BVH = bilateral vestibular hypofunction, UVH = unilateral vestibular hypofunction, HCs = healthy controls, ACC = acceleration, RMS = root mean square, MV = mean sway velocity, SD = standard deviation.

**TABLE 5:** Subgroup Differences on the Tandem Romberg Test

	One-way ANOVA							Post hoc comparisons^b^		
	BVH group ( $\text{n}=20$) (mean±SD)	UVH group ( $\text{n}=11$) (mean±SD)	HC group ( $\text{n}=31$) (mean±SD)	$F$	$p$-value	$p$-value^a^	$\eta ^{2}$	BVH vs. UVH groups	UVH vs. HC group	BVH vs. HC groups
								$p$-value	$p$-value	$p$-value
Tandem Romberg test (eyes open)										
Head										
Medio-lateral axis										
ACC (maximum), m/s^2^	0.86±0.58	0.54±0.21	0.47±0.19	7.157	0.002	0.020	0.195	0.070	0.879	0.002
ACC (mean), m/s^2^	0.16±0.08	0.13±0.04	0.11±0.04	5.053	0.009	0.113	0.146			
ACC (RMS), m/s^2^	0.21±0.11	0.16±0.06	0.14±0.05	5.735	0.005	0.064	0.163			
MV, m/s	1.65±0.88	1.33±0.46	1.13±0.38	4.671	0.013	0.132	0.137			
Cranio-caudal axis										
ACC (mean), m/s^2^	0.07±0.05	0.04±0.01	0.05±0.02	4.398	0.017	0.199	0.130			
Pelvis										
Medio-lateral axis										
ACC (maximum), m/s^2^	0.40±0.23	0.35±0.17	0.28±0.09	3.488	0.037	0.444	0.106			
ACC (mean), m/s^2^	0.08±0.04	0.07±0.03	0.06±0.02	4.413	0.016	0.196	0.130			
ACC (RMS), m/s^2^	0.11±0.05	0.09±0.04	0.08±0.02	4.394	0.017	0.200	0.130			
MV, m/s	0.85±0.41	0.76±0.41	0.61±0.18	3.533	0.036	0.168	0.107			
Ankles										
Antero-posterior axis										
ACC (maximum), m/s^2^	0.81±0.46	0.50±0.45	0.51±0.22	5.065	0.009	0.112	0.147			
ACC (mean), m/s^2^	0.10±0.04	0.07±0.03	0.07±0.04	4.402	0.017	0.198	0.130			
ACC (RMS), m/s^2^	0.13±0.06	0.09±0.04	0.09±0.05	4.334	0.018	0.210	0.128			
MV, m/s	0.99±0.44	0.71±0.36	0.67±0.41	3.723	0.030	0.360	0.112			
Tandem Romberg test (eyes closed)										
Head										
Medio-lateral axis										
ACC (mean), m/s^2^	0.31±0.21	0.34±0.24	0.21±0.08	3.570	0.035	0.418	0.115			
ACC (RMS), m/s^2^	0.42±0.32	0.47±0.35	0.28±0.13	3.200	0.048	0.582	0.104			
MV, m/s	3.06±2.20	3.56±2.68	2.08±0.76	3.641	0.033	0.393	0.117			
Antero-posterior axis										
ACC (mean), m/s^2^	0.24±0.22	0.41±0.53	0.16±0.07	3.743	0.030	0.359	0.120			
ACC (RMS), m/s^2^	0.32±0.33	0.51±0.61	0.21±0.10	3.413	0.040	0.481	0.110			
MV, m/s	2.41±2.30	4.44±6.06	1.61±0.71	3.867	0.027	0.322	0.123			

^a^ =Bonferroni-corrected $P$ value, $^{}\eta ^{2}$ = eta square,

^b^ = Scheffe-corrected post hoc $t$ test, BVH = bilateral vestibular hypofunction, UVH = unilateral vestibular hypofunction, HCs = healthy controls, ACC = acceleration, RMS = root mean square, MV = mean sway velocity, SD = standard deviation.

As shown in Table 4, in the eyes-closed portion of the Romberg test, participants with VH exhibited notable differences in head, pelvis, and ankle movements compared with HCs. Specifically, BVH participants exhibited higher maximum ACC, mean ACC, RMS of ACC, and MV along the ML axis. Additionally, their ankle movement exhibited a higher maximum ACC along the CC axis. Following Bonferroni correction, significant differences persisted in head movements along the ML axis, as well as in pelvic movements, for the maximum ACC and RMS of ACC.

As shown in Table 5, in the eyes-open portion of the tandem Romberg test, participants with VH exhibited different head and pelvis movement patterns from HCs. BVH participants exhibited higher maximum ACC, mean ACC, RMS of ACC, and MV along the ML axis. Moreover, BVH participants exhibited substantial head movement with notably higher mean ACC along the CC axis. They also exhibited higher maximum ACC, mean ACC, RMS of ACC, and MV along the AP axis for ankle movement. After Bonferroni correction, only head movement along the ML axis in terms of maximum ACC retained its statistically significant difference.

Further post hoc comparisons revealed that the observed differences were primarily between the BVH and HC subgroups, highlighting the pronounced impact of BVH on these movement parameters.

## IV. Discussion

In this cross-sectional study, we used wearable sensors to monitor individual movement kinematics of the head, pelvis, and ankles in the Romberg and tandem Romberg tests to examine the instability induced by VH. A total of 144 parameters, including the maximum and mean ACC, RMS of ACC, and MV along three axes, were analyzed. Among these, a subset of parameters emerged as statistically significant differentiators between healthy adults and those with VH. It is worth noting that the conventional approach for the Romberg test often involves the use of a compliant memory foam surface during the feet-together condition to increase difficulty. However, in this study, to ensure simplicity and accessibility in various clinical settings, the Romberg test was conducted on a firm surface. This decision was made to facilitate ease of administration, considering the limited availability of compliant memory foam surfaces for testing purposes. Furthermore, the inclusion of the tandem Romberg test aimed to simulate reduced proprioceptive input, similar to standing on a compliant surface. By incorporating both tests, a comprehensive assessment of balance control under various conditions was performed. The results of this study indicate that the wearable IMUs can quantify movement in the Romberg and tandem Romberg tests to explore the extent of movement in adults with VH.

### A. Addition of Wearable IMUS to Romberg Test and Tandem Romberg Test

The methods of applying the Romberg test vary [6], and positive test results also vary (for example, a fall [35], a sway [36], or needing to open the eyes or move the limbs within a set time interval [37], [38], [39]). Herein, we propose a novel method of adding instrumentation to the Romberg test to characterize body movement in individuals with VH and to quantify the test results. The instrumented eyes-closed portion of the Romberg test could differentiate between participants with VH and HCs.

This study also demonstrated the feasibility of using the IMU-based instrumented approach in the tandem Romberg test. Longridge and Mallinson [11] reported that the tandem Romberg test provided no value in diagnosing vestibular disease. Additionally, performance on the tandem Romberg test differed significantly between the young (< 50 years old) and old (≥50 years old) cohorts. The authors assumed that age effects on the tandem Romberg test may overshadow the ability to detect a vestibular disease [11], as age may affect balance. Indeed, the proportion of the general population with vestibular vertigo increases with age [1]. In the elderly population, VH is the leading cause of balance problems [40], [41]. Therefore, we applied an age cutoff of 50 years in our study, enrolling only adult participants less than 50 years old. Compared with the typical tandem Romberg test, the proposed instrumented tandem Romberg test effectively differentiated participants with VH from HCs.

The findings support the initial hypothesis that IMU-based Romberg and tandem Romberg tests can provide valuable insights into balance impairments associated with VH. The significant differences observed in kinematic measures between VH participants and HCs indicate the sensitivity of the IMU system in detecting abnormal body motion during balance tasks. The study also highlights the advantages of using instrumented tests over traditional Romberg tests that rely on visual observation. By quantifying balance measures using IMUs, we overcame the subjectivity and variability inherent in visual assessments. This objective and quantitative approach allows accurate and consistent evaluation of balance performance.

### B. Kinematic Features During Standing Balance Tests for HCS and Participants With VH

Postural balance relies on integrating the visual, proprioception, and vestibular systems, which tend to be interdependent and supportive [6], [42]. Losing two or more of these systems may lead to imbalance or a fall, but a healthy individual can maintain posture when only one system is lost [6]. Thus, in the eyes-open portion of the Romberg test, we found no significant differences between the VH and HC groups. The participants with VH could maintain an upright stance by relying on visual and proprioception inputs. In the eyes-closed portion of the Romberg test, HCs lost only visual input; in contrast, participants with VH lacked both visual and vestibular input and thus exhibited significant body sway along the ML axis. This result is consistent with the literature.

HCs effectively maintained balance through the integration of two inputs in each of these tests—proprioception and vestibular inputs in the eyes-closed Romberg test and visual and vestibular inputs in the eyes-open tandem Romberg test. Conversely, participants with VH relied on a singular input for each test—proprioception input for the eyes-closed Romberg test and visual input for the eyes-open tandem Romberg test. A comparison of the original data between the eyes-closed portion of the Romberg test and the eyes-open portion of the tandem Romberg test indicated similar results. However, it is worth noting that the discrepancy in these findings—particularly the observed statistical significance only in the head and pelvic movement parameters along the ML axis in the eyes-closed Romberg test after Bonferroni correction—may indicate a potential weakening rather than complete loss of proprioception in the tandem Romberg test. Compared with HCs, participants with VH exhibited a higher degree of body sway along the ML axis. These results confirm that monitoring and maintaining optimal balance requires at least two inputs. Additionally, the experimental results support our hypothesis. The IMU-based instrumented Romberg and tandem Romberg tests, which evaluate balance, can provide objective data to improve the reliability and validity of the traditional Romberg and tandem Romberg tests.

Standing with one foot in front of the other (as in the tandem Romberg test) disrupts proprioception, which is the primary source of postural information for an individual with VH in the eyes-closed portion of the test. In this portion of the tandem Romberg test, participants with VH experienced a reduction in three sensory inputs, while HCs experienced a reduction in two sensory inputs. Both groups exhibited a lack of balance (e.g., body sway), and no significant distinctions were noted following the Bonferroni correction. This finding indicated that loss of two or more sensory inputs significantly contributed to pronounced body sway in participants with VH and HCs.

The RMS of ACC has been used to assess gait and balance [19], [43]. The RMS values along the ML axis were elevated in balance-impaired individuals [44]. Similarly, the RMS ratio along the ML axis was associated with walking balance [43]. In the present study, we used wearable sensors to capture the ACC of body movements and calculated the RMS of ACC for the Romberg test and tandem Romberg test. We found that individuals with VH exhibited a large RMS of ACC along the ML axis than HCs.

The results indicate the clinical implications and advantages of using IMU-based assessments in evaluating balance impairments. The ability of IMUs to objectively quantify kinematic characteristics allows clinicians to monitor balance performance more comprehensively and track the progress of individuals undergoing vestibular rehabilitation [45], [46]. Furthermore, the ability to assess multiple body parts, including the head, pelvis, and ankles, can clarify balance control mechanisms and facilitate targeted interventions.

### C. Subgroup Analysis

Our analysis pinpointed a significant differentiation between HCs and subgroups of participants with BVH in terms of kinematic parameters related to head movement—specifically the metric of maximum ACC. Therefore, tracking head movement is essential for assessing posture stability and balance control in patients with vestibular deficits [21], [47], [48]. Comparison of HCs and participants with UVH revealed no significant differences in parameters on the eyes-open or eyes-closed portions of the Romberg test or the eyes-open portion of the tandem Romberg test. Participants with UVH may have effectively compensated for their unilateral vestibular deficit. Further research is needed to investigate the characteristics of posture control in adults with UVH.

The subgroup analysis comparing BVH participants to HCs and UVH participants provided additional insights. The significant differences observed in kinematic measures between BVH participants and HCs suggested more pronounced balance deficits in the BVH subgroup. This information can be useful for tailoring treatment approaches and interventions for individuals with different types of VH.

### D. Limitations

This study had limitations that should be acknowledged. First, it was a cross-sectional study, and the statistical analyses of demographic characteristics were conducted post hoc. Second, the sample size—particularly for the UVH and BVH subgroups—was small. Nonetheless, the calculated effect sizes using eta squared indicated meaningful trends and differences. Additionally, Cohen’s d was employed to compare the two groups, revealing effect sizes that complement the interpretation of results. However, future studies with larger sample sizes are needed to further enhance the robustness and generalizability of the conclusions derived from the analysis. Third, it is important to note that these tests are indirect measures of the vestibular system. Patients with foot deformities; musculoskeletal problems affecting the ankle, knee, hip, or spine; peripheral neuropathy; or other neurological conditions may score poorly on these tests, even with an intact vestibular system. Therefore, it is essential to consider the effects of these factors when interpreting the results and applying the tests in clinical practice.

In future studies, we plan to address these limitations by recruiting a more diverse range of participants from different age groups, including individuals over the age of 50 years. This approach will allow us to determine the feasibility and discriminative validity of these tests in a broader population. Additionally, we intend to explore more advanced kinematic tests, such as Timed Up and Go, to provide comprehensive ambulatory indexes for clinical assessment.

Furthermore, we are considering the incorporation of angular velocity measures in our future research to more comprehensively analyze balance performance. Advanced filtering approaches will also be investigated to separate dynamic acceleration and gravity for evaluating the balance of individuals with VH, including the Butterworth low-pass filter [49], inclination angles [50], and sensor fusion [51]. By incorporating these measures and signal processing techniques, we can enhance the clinical applicability of our findings.

## V. Conclusion

The IMU-based instrumented Romberg and tandem Romberg tests are valuable objective tools for identifying individuals with VH. Particularly noteworthy are the head and pelvic movements along the ML axis of maximum ACC, as well as head movement in the RMS of ACC in the eyes-closed portion of the Romberg test, which effectively distinguish healthy adults from those with VH. Patients with BVH are likely to exhibit noticeable swaying along the ML axis during the eyes-closed Romberg test or eyes-open tandem Romberg test. Notably, the maximum ACC of head movement in the ML axis stands out as a pivotal indicator for differentiating between BVH and HCs. These findings have potential utility as a reference for VH screening in primary care clinics lacking access to specialized vestibular function tests.
